# Eating Competence and Aspects Related to a Gluten-Free Diet in Brazilian Adults with Gluten-Related Disorders

**DOI:** 10.3390/nu14142815

**Published:** 2022-07-08

**Authors:** Pâmela Mayara de Oliveira, Renata Puppin Zandonadi, Amanda Moreira Veloso Cutrim, Eduardo Yoshio Nakano, Fabiana Lopes Nalon de Queiroz, Raquel B. A. Botelho, Ariana Saraiva, António Raposo

**Affiliations:** 1 Department of Nutrition, Faculty of Health Sciences, Campus Universitário Darcy Ribeiro, University of Brasilia, Brasilia 70910-900, Brazil; amvc.amanda@gmail.com (A.M.V.C.); fabinalon@hotmail.com (F.L.N.d.Q.); raquelbotelho@unb.br (R.B.A.B.); 2 Department of Statistics, Campus Universitário Darcy Ribeiro, University of Brasilia, Brasilia 70910-900, Brazil; eynakano@gmail.com; 3 Department of Animal Pathology and Production, Bromatology and Food Technology, Faculty of Veterinary, Universidad de Las Palmas de Gran Canaria, Trasmontaña s/n, 35413 Arucas, Spain; ariana_23@outlook.pt; 4 CBIOS (Research Center for Biosciences and Health Technologies), Universidade Lusófona de Humanidades e Tecnologias, Campo Grande 376, 1749-024 Lisboa, Portugal

**Keywords:** eating competence, questionnaire validation, gluten-related disorders, gluten-free diet

## Abstract

This cross-sectional study aims to assess eating competence (EC—an intra-individual approach to food, behaviors, and attitudes related to food) and aspects related to a gluten-free diet (GFD) in Brazilian adults with gluten-related disorders (GRDs). The research was conducted using an online survey with a self-reported instrument consisting of 40 items, organized into three parts: (I) Socioeconomic and demographic data; (II) the Brazilian version of the Eating Competence Satter Inventory (ec-SI2.0™BR); and (III) questions about adherence and difficulties in following the gluten-free diet. EC was measured by the ecSI2.0™BR instrument, with scores ≥32 were considered competent eaters. The instrument was applied nationwide through the GoogleForms^®^ platform from 14 February 2022 to 30 March 2022. The publicity for the recruitment was supported by Brazilian celiac local and national associations (Acelbras and Fenacelbra), pages of food services or personal pages of tips and posts about gluten-related disorders, and specialized stores that offer gluten-free foods. The recruitment occurred through social networks (emails, Facebook groups, WhatsApp, and Instagram). A total of 1030 Brazilians with GRDs answered the questionnaire. Most participants were female, aged 40 years or older, with an income >R$3000, and a high education level. The main difficulty regarding adherence to GFD was the high cost of gluten-free foods. Individuals younger than 40 years old had lower EC scores, with no differences between men and women. Increasing socioeconomic status, schooling, and culinary practices increased the total score. Participants who “never/almost never” felt socially judged because their diet had higher scores for total EC. Competent eaters GRD individuals (EC ≥ 32) were mostly individuals aged ≥40 y/o; with income > R$3000; following a GFD; satisfied with purchased gluten-free products; consuming gluten-free products prepared at home, mainly by themselves; who do not feel judged because of the GRD and who feel that they can live a normal life with GRD. Our study showed that individuals who strictly adhere to the GFD have higher scores on eating competence than those who sometimes follow the treatment.

## 1. Introduction

Gluten-related disorders (GRD) are a group of diseases that share gluten ingestion as an etiologic factor. The main GRD are celiac disease (CD), non-celiac gluten sensitivity (NCGS), gluten ataxia, dermatitis herpetiformis (DH), and wheat allergy (WA) [1,2]. It is estimated that about 10% of the world’s population present GRD and, currently, the only safe and available treatment is a gluten-free diet (GFD) [3,4]. The GFD implies excluding food containing wheat, rye, barley, and their by-products and, in some cases, oats [5,6].

The diet of people with GRDs should be not only gluten-free (GF) but also provide nutritional and energy supply, playing an important role in the individual’s health [7,8]. In some studies, an imbalance of GFDs is reported, characterized by low consumption of vegetables, legumes, and fruits and increased consumption of high glycemic index food. Gluten-free products are commonly rich in refined starches and fat since it is a challenge to balance the sensory, technological, and nutritional quality of gluten-free food [7,9,10,11,12].

Unbalanced GFD can result in metabolic consequences, nutrient deficiencies, and weight gain. The inadequacy of GFD is expected when the individual is not adequately oriented about food (choices and consumption) and a healthy diet since there is often an excess of lipids and refined starches in gluten-free food to compensate for the gluten withdrawal. GF products tend to be more expensive, with a lower nutritional and sensory quality than GF food, jeopardizing GRD treatment and health maintenance [11,13,14,15].

In addition to these challenges, individuals face social difficulties, as they cannot consume the same preparations as non-GRD patients; difficulties in accessing and availability of GF foods; accessibility to food services that provide safe GF foods [16,17,18,19]. Therefore, following a GFD is challenging due to the lack of information and guidance on healthy GF meal preparation, high food costs, and the need for health support and information. Adjusting long habits of consuming food prepared with gluten to a new food consumption style, combined with a lack of cooking skills, may favor GFD transgression impairing the GRD people’s health [16,20,21,22].

Despite advances in knowledge about food, nutrition, and their relationship with health, there is a continuous increase in diseases linked to poor diet [23], mainly in individuals with dietary restrictions as in GRDs [24,25,26,27]. GRD can increase the risk for diabetes mellitus, weight disbalance, fatty liver, metabolic syndrome, cardiovascular risk, and cancer [28,29,30,31,32,33]. It also can lead to nutrient deficiencies such as iron, folate, vitamin B12, vitamin D, calcium, copper, zinc, and vitamin B6 [1,28] evidencing the need for improvements in the diet of these individuals.

The act of eating concerns the way of relating to food in different spheres. Food choices go beyond biological and nutritional needs, and in the case of GRDs, it is also conditioned to the limitations imposed on the individuals’ food consumption as a form of treatment. Food consumption is determined by choices whose complexity involves biological, social, cultural, economic, and psychological factors, as well as access to food. Sometimes food choices are conscious, but they can also be automatic, habitual, and subconscious [34]. Therefore, eating is considered a complex process draft of learned behavior, social expectations, acquired tastes, attitudes, and feelings about eating and food [35]. On this path, linked to health promotion and better diet quality, came up the concept of eating competence (EC), defined as an intra-individual approach to food, behaviors, and attitudes related to food, with positive biopsychosocial results according to the Eating Competence Model proposed by Satter (ecSatter) [36,37].

The EC concept describes eating practices based on four components: Eating Attitude—having a positive and relaxed attitude about food and eating; Food Acceptance—having an interest in food, experimenting with unfamiliar food, leading to dietary variety; Internal Regulation—being naturally attentive to internal signals of hunger, appetite, and satiety; and Contextual Skills—having resources to manage the food context [37]. According to the ecSatter model, individuals are better able to deal with food when they have self-confidence concerning food choices and are willing and open to new experiences with food, achieving a balance between desires, choices, and quantities to be ingested.

EC is associated with better diet quality [38] and greater skills in handling and managing own food [39], indispensable factors for individuals with dietary restrictions. Findings also reveal that EC is linked to psychological and behavioral aspects, such as greater satisfaction with body weight and lower frequency of behaviors associated with eating disorders [40,41,42].

The EC and its four components can be measured through the Eating Competence Satter Inventory (ecSI2.0™), which evaluates individuals by classifying them into two groups, competent and non-competent eaters [43,44]. This instrument has already been validated in Brazilian-Portuguese but tested only with the general population [45,46].

There is an urgent need for guidance and the knowledge necessary for choosing foods for an individual to have adequate adherence to the GFD. Given the characteristics of GRD and its treatment totally associated with the individual’s diet and the increase in the development of chronic diseases, especially in individuals with dietary restrictions, this study presents two main hypotheses: (i) Individuals with GRDs have greater EC than the general Brazilian population; (ii) GRD individuals who adhere to a GFD have greater EC than those who do not adhere to treatment. It is worth mentioning that the GFD forces them to pay attention to their diet, potentially making them more likely to opt for a healthier diet. However, food access, preparation, understanding, and quality difficulties may impair GFD adherence. Therefore, the primary objective of this study was to evaluate EC in Brazilian adults with gluten-related disorders. This study also aimed to assess the adherence to a gluten-free diet and aspects related to food selection, purchase, preparation, and difficulties in following a GFD, aspects potentially associated with eating competence.

## 2. Materials and Methods

### 2.1. Study Design

This quantitative cross-sectional study was performed using a convenience non-probability sampling method by online snowball recruitment in the Brazilian adult population with GRD after approval by the Research Ethics Committee (CAAE 24415819.2.0000.8101). This method was used considering that we were facing the COVID-19 pandemic in Brazil during the data collection period, limiting access to face-to-face research. Moreover, studies have shown that snowball sampling via social media is an effective and efficient way to recruit study participants. This method makes it possible to achieve increased sample size and decreased time to completion, as well as a reduction in application cost. An instrument was constructed based on previously validated questionnaires and one constructed and validated for this research, as follows [47,48].

### 2.2. Instrument Construction

The research instrument was organized into three parts: (I) Socioeconomic and demographic data; (II) the Brazilian version of the Eating Competence Satter Inventory (ecSI2.0™BR); and (III) questions on adherence and difficulties in following GFD and aspects related to food selection, purchase, preparation.

The first part of the instrument was used to characterize the population sample and then allow for better data analysis, including questions about sex, age, income, education, and area of residence (using the Brazilian Institute of Geography and Statistics (IBGE) questions as a reference [49]) and if the participant has any other restrictions besides gluten. The second part of the instrument was used to evaluate eating competence among the participants, using the Brazilian Satter eating competence inventory (ecSI2.0™BR) [50]. The ecSI2.0™BR is composed of 16 questions previously validated for online use among the Brazilian adult population [51], and it is available online at https://www.needscenter.org/ (accessed on 5 April 2021) upon application approval [46]. The use of ecSI2.0™BR for this study was previously authorized by NEEDs Center. To classify the individual as a competent eater, a minimum total score of 32 was considered as the cut-point [44,46]. The third part was constructed and validated for this study. It was composed of questions on adherence and difficulties in following the GFD, and aspects related to food selection, purchase, and preparation since these aspects may impair GFD adherence and impact EC.

The items of the third part of the instrument were established through an extensive literature review aiming to cover the main factors GRD individuals face with GFD that could potentially impact EC. After items selection, validation of these questions was performed first by an expert panel (*n* = 22) using the Delphi method [51] to evaluate the pertinence and semantic evaluation. When an agreement of at least 80% was reached among experts per item [51,52], the reproducibility (reliability) and internal consistency were tested in a pilot study with a small convenience sample of 30 GRD individuals. At this step, the internal consistency and reproducibility showed good agreement (kappa > 0.6 and absolute agreement ≥ 70%) [53]. At the end, the third part of the instrument related to the GFD was validated and composed of the following questions: (1) Do you follow a gluten-free diet?; (2) What is the main reason you follow a gluten-free diet?; (3) What helps you maintain your gluten-free diet?; (4) What are the difficulties you have in following a gluten-free diet?; (5) How often do you buy gluten-free products?; (6) Are you satisfied with the gluten-free products you buy?; (7) If you buy sometimes or never buy special gluten-free products (e.g., gluten-free bread, gluten-free pasta, gluten-free cake, etc.), why?; (8) How often do you consume gluten-free products prepared at home?; (9) If you consume gluten-free products prepared at home, who prepares them?; (10) If you do not consume special gluten-free products (e.g., gluten-free bread, gluten-free cake, gluten-free cookies, etc.) prepared at home, why?; (11) Do the people you live with help you to have a gluten-free diet?; (12) How frequently do you feel socially judged and/or disapproved of for having this gluten-related disorder?; (13) How frequently do you feel I can’t live a normal life because of this gluten-related disorder?; (14) How often do you feel that you cannot eat meals with your colleagues, friends and/or family at meetings or events?

**Figure 1:**
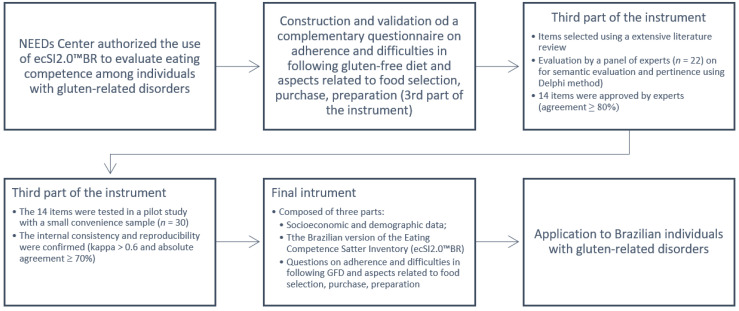
Steps followed until the final instrument application.

The steps followed until the final instrument application are summarized in Figure 1.

### 2.3. Settings and Participants

After confirming the reproducibility and internal consistency of the questions regarding the difficulties individuals face with GRDs and GFD, the ecSI2.0™BR was added to complete the instrument to apply nationwide to GRD individuals. The application of the instrument at the national level was made virtually through the GoogleForms^®^ platform starting on 14 February 2022, until 30 March 2022. The publicity for participants’ recruitment was carried out with the support of Brazilian celiac local and national associations (Acelbras and Fenacelbra), as well as pages of food services and specialized stores that offer GF foods or personal pages of tips and posts about gluten-related disorders, being disseminated through social networks (emails, Facebook groups, WhatsApp, and Instagram).

The inclusion criteria for participating in the research were: (i) Being Brazilian aged 19 years or older; (ii) having at least one gluten-related disorder diagnosed by a physician (self-reported); (iii) accepting to participate in the study after reading and signing the informed consent term.

The sample calculation predicted a minimum of 381 respondents nationwide, respecting the minimum quotas for each geographic region. The minimum size of the national sample considered was the proportion of the Brazilian population by region, according to the last demographic census [54]: North = 8.34%, North-East = 27.8%, Midwest = 7.36%, South-east = 42.2% and South = 14.3%. A standard deviation of 8.9 was considered in the ecSI2.0™BR score [51] to obtain the minimum sample required with a margin of error of 3.3 points (10% of the expected score) and a confidence of 95%. Under these conditions, the minimum number of individuals in the Brazilian Midwest region (a less populated region, with 7.36% of the Brazilian population) must be 28 individuals, resulting in a minimum national sample of 28/7.36% = 381 respondents, respecting the minimum quotas for each geographic region (North = 32, North-East = 106, Midwest = 28, South-East = 161 and South = 54).

### 2.4. Bias

Some methodological limitations should be pointed out since using an online snowball survey, respondents gathered through non-random probability, make it difficult to generalize the results, particularly those related to Internet access. We used this method because it is less costly and less invasive, requiring less effort and time for the researchers and participants than a face-to-face interview [47,48]. Face-to-face interviews could also cause difficulties in reaching participants due to geographical limitations [55] and the COVID-19 pandemic (in a course during data collection). A study comparing online and paper-pencil methods found that the ecSI^TM^ scores did not differ according to the method of survey completion [43]. Moreover, data from the Brazilian population showed that three out of four Brazilians have Internet access, and about 93.2% of the adult population have a cellphone, a primary tool used to access the Internet [56]. Therefore, the online self-reported method was efficient for data collection during the pandemic, despite it was not possible to control the response numbers and stratification to achieve socioeconomic and demographic representativeness.

### 2.5. Statistical Analysis

The scores of the ecSI2.0™BR (and its components) were described in terms of means and standard deviations (SD) for quantitative variables and frequencies and percentages for categorical responses, and interclass correlation coefficient (ICC) for reproducibility of the difficulty questionnaire presented by individuals with GRDs. Student’s *t*-test and Analysis of Variance (ANOVA) followed by Tukey’s post-hoc tests were used to compare the scores of ecSI2.0™BR with the variables of interest. The Kolmogorov–Smirnov test verified the normality assumption. Results of categorized eating competence (EC ≥ 32) were described in terms of frequencies and percentages, and Pearson’s chi-squared tests verified its association with the variables of interest. All tests were performed considering bilateral hypotheses and a 5% significance level. The analyzes were performed using IBM SPSS (IBM SPSS Statistics for Windows, IBM Corp., Armonk, NY, USA) version 22.

## 3. Results

Of the 1133 participants who accessed and answered the online questionnaire, 103 responses were considered invalid as they did not meet the inclusion criteria (having some gluten-related disorders and age from 19 years). The final sample consisted of 1030 individuals with valid answers. Table 1 presents the socioeconomic characteristics of the participants and the EC scores. Most participants were female (*n* = 986; 95.7%), aged 40 years or older (*n* = 531; 51.5%); with a high level of education (*n* = 465; 45.1% graduate and *n* = 407; 39.5% undergraduate) and with family income > R$3000 (R$1 = USD 0.19) (*n* = 702; 68.1%) (Table 1). As for the main reason for following a gluten-free diet, 781 responded that they followed GFD because of celiac disease (CD) (Table S1). Some participants (*n* = 139) had simultaneously two or more diseases among wheat allergy, celiac disease, dermatitis herpetiformis, and non-celiac gluten sensitivity. Most of them (*n* = 50; 35.9%) presented celiac disease and dermatitis herpetiformis; wheat allergy and non-celiac gluten sensitivity (*n* = 25; 17.9%); or wheat allergy and celiac disease (*n* = 16; 11.5%). Similar results were observed performing the analysis on CD and non-CD crossing the scores of the ecSI2.0™BR and sociodemographic data (Supplementary Tables S2 and S3).

The mean total EC score of individuals with GRDs was 31.88 ± 7.97 for females and 32.61 ± 7.70 for males, with no significant difference between the two groups (*p* > 0.05). Individuals aged ≥40 y/o had a better total EC score and contextual skills than those aged up to 40 years (*p* < 0.05) (Table 1). Individuals with the highest educational level presented higher scores for food acceptance but did not differ in total EC and other parameters. Individuals with the highest income level had higher scores for food acceptance than the lowest income level, but it did not differ in other EC parameters. Individuals with other restrictions besides gluten had higher scores for contextual skills than those only with GRD and did not differ in total EC and other parameters. Participants following a GFD had higher scores (except for food acceptance) in total EC and their components than those who sometimes follow the GFD. EC did not differ among individuals considering the frequency of buying GF products (*p* > 0.05). However, among those who purchase GF products, those satisfied with these products have better scores in all EC parameters than those not satisfied with the products.

The highest the frequency of consumption of GF products prepared at home, the highest the EC scores. Most of the participants (*n* = 902) used to prepare their GF products. Individuals who prepare their GF meals present higher scores for all EC parameters than those with which another person prepares their GF meals at home. Moreover, participants supported by people living with to adhere to a GFD presented the highest EC scores.

People that fell to live an everyday life because of the GRD and do not feel socially judged and/or disapproved had the best scores to total EC and its parameters. Those who feel that they cannot eat meals with their colleagues, friends and/or family at meetings or events presented lower EC scores.

GRD individuals considered competent eaters (EC ≥ 32) were most individuals aged ≥40 y/o; with income > R$3000; following a GFD; satisfied with GF products purchased; consuming GF products prepared at home, mainly by themselves; who do not feel judged because of the GRD and who feel that they can live a normal life with GRD. In our sample, individuals with celiac disease are more competent eaters than non-celiac individuals (*p* = 0.023). They also had higher scores for eating attitude (*p* = 0.003) and total EC score (*p* = 0.029) than non-celiac individuals.

Concerning the diet, the three main reasons for not buying or rarely buying GF products are: Because they are too expensive (36.9%); followed by preparing my GF products at home (24.3%); and stores are difficult to access (14.1%). The main reasons for not consuming special products prepared at home are that: The ingredients are expensive (38.6%); followed by “I don’t have time to cook” (21.2%) and “I don’t easily find the ingredients to prepare these recipes” (20.7%); the high price of these foods (29.5%) is also the main difficulty in following a GFD; “I feel different from others following a gluten-free diet”; the second biggest difficulty (11.1%) and the third biggest reason was the lack of time to prepare meals (9.0%) (Supplementary File—Table S1).

Most participants (46.9% of total responses for this multiple answers questions or 82.7% of respondents) report that what contributes to maintaining a GFD is the preparation of GF food at home. The second biggest reason is the financial availability to purchase gluten-free products (18.0%), followed by easy access to food (14.2%) (Supplementary File—Table S1). It is important to highlight that in the question “What helps you maintain your gluten-free diet?” 852 patients declared “I prepare gluten-free foods at home”, while in *question* “If you buy sometimes or never buy special gluten-free products (e.g., gluten-free bread, gluten-free pasta, gluten-free cake, etc.,), why?” only 136 patients declared “I prepare my gluten-free products at home”. This occurred because in the first question, all respondents were invited to mark an option; in the second, only the participants that “buy sometimes or never buy special gluten-free products” (*n* = 270) were asked to answer the question.

## 4. Discussion

This study is the first in which the ecSI2.0™BR instrument has been used to assess the EC of Brazilian adults with gluten-related disorders. Of 1030 Brazilian adults, most were female, with a high level of education and income, similar to those found in the study on EC in the general Brazilian population in which most of the participants were female (*n* = 1353; 74.75%), with a high level of education (*n* = 882; 48.72% were graduates) and family income > R$5000 (*n* = 1239; 67.9%) [46]. The sample predominantly composed of females reinforces the greater concern of women regarding health and a tendency to participate in health research more than males [1,18,57,58,59]. A study conducted in Brazil with GRD individuals [18] also showed more responses from CD females (95%); it could also be explained by the prevalence of gluten-related disorders that is higher in females than in males (2:1 to 3:1) [1,18,57,58,59].

The present study demonstrated that celiac disease (CD) is the main reason for following a GFD (75.8%). This result probably occurred because the prevalence of wheat allergy and dermatitis herpetiformis is lower than CD. In Brazil, the non-celiac gluten sensitivity is still mostly “self-diagnosed”, and those not diagnosed by physicians were not invited to participate in the study. Also, in Brazil, the national and local celiac associations [60] constantly reinforce to celiacs the importance of GFD to avoid harmful consequences of the CD and they helped us disseminate the search.

The mean total EC score of individuals with GRDs for females (31.88 ± 7.97) and males (32.61 ± 7.70) was higher than that found in the general Brazilian population (females 30.26 ± 9.02 and males 29.94 ± 8.52) [46]. This result confirms our first hypothesis that individuals with GRDs have greater EC than the general population, probably because their treatment forces them to pay more attention to food and diet. In both studies, there was no difference in total EC between genders. Considering the validated cut-off value of ≥32 [44,61], about 56% (*n* = 578) of the sample were classified as competent eaters, higher than that found in a previous study with the general Brazilian population (47.73%) [46], probably due to the nature of GRD in which individuals must have more attention to food choices, purchases, and preparation [19,62,63]. In the present study, competent eaters (EC total score ≥ 32) were primarily individuals over 40 years of age with higher educational levels and income.

Individuals with the highest income level had higher scores for food acceptance than the lowest income level, but it did not differ on other EC parameters. The high cost of GF could explain it, where people with higher income have access to GF products with the best quality, influencing food acceptance. Results on the main difficulties in buying GF products (prices of GF food—36.9%, preparing GF food at home—24.3%, and stores with difficult access—14.1%) reinforce this hypothesis since people with higher incomes have more access to purchase and access food and prepare food at home [64,65].

Participants following a GFD had higher scores on total EC and their components, except for food acceptance, than those who sometimes follow the GFD. Once GF food tends to present poor sensory quality, it can impair food acceptance of those who consume it, even if not regularly. Individuals that adhere to strict GFD are more aware of the importance of understanding and following the treatment to avoid harmful consequences on health and quality of life [15,66,67,68], probably impacting the EC and its components. This result partially confirms our second hypothesis that GRD individuals who adhere to a GFD have greater EC than those who do not adhere to treatment. In our sample, CD individuals are more competent eaters than non-CD individuals and had higher eating attitudes and total EC scores, probably due to celiacs need to follow a lifelong strict GFD. Considering that GFD must be strictly followed by celiacs every day to avoid negative consequences of untreated CD, individuals who answered sometimes follow a GFD (*n* = 41) were mostly non-celiacs (*n* = 30; 75%) who did not need to follow a strict GFD, only the celiacs (*n* = 11) should be considered not following the treatment. Those who answered that they never follow the GFD do not show significant differences from the GFD followers. However, it could be explained by the low number of individuals (*n* = 7) that never follow the GDF, compromising the statistical significance. Comparing the proportion of respondents, about 99% (*n* = 766) of individuals with CD answered always following GFD, higher than non-celiacs (87%; *n* = 249) always following GFD (Supplementary Tables S2 and S3). These patients adhere to GFD differently by definition, and recent data suggest that adherence to GFD is not so strict for non-celiac patients with GRD. However, the percentage of non-celiac is high (>85%) since in Brazil, we adopt the GFD for these patients as well as in the case of celiacs.

Food prices were reported as the main difficulty in following the GFD since GF products are more expensive and can cost up to 267% more than their gluten-containing counterparts [69]. The high prices of GF products are still a great concern to GRD individuals, as demonstrated in the present study (Supplementary File—Table S1) and others previously published [70,71].

EC did not differ among individuals considering the frequency of buying GF products (*p* > 0.05). However, among those who purchase GF products, those satisfied with these products have better scores in all EC parameters than those not satisfied with the products. Dissatisfaction with GF products can lead to low adherence to GFD and complications such as low self-regulation, self-efficacy, social fear, depression or frequent lapses in self-control, symptoms directly linked to low scores EC, and a worse quality of life [35,72,73].

Those who frequently consume GF products prepared at home presented the highest EC scores, probably due to the attention given to the ingredients choices and meals’ preparation. The attention given to diet to avoid gluten ingestion probably makes most participants (*n* = 902) prepare their meals. Individuals who prepare their GF meals present higher scores for all EC parameters reinforcing the importance given by participants to GFD. Our results showed that the main reason for maintaining the GFD was preparing GF food at home (46.9% of total responses for this multiple answers questions or 82.7% of respondents). Considering this proportion of respondents is well in line with the result of Rodrigues et al. [71] in which 88.6% of the GF recipes were prepared by patients.

Participants supported by people living with to adhere to a GFD presented the highest EC scores since family support has a positive role in adherence to GFD and coping with GRDs [18,74,75]. People who fell to live a normal life because of the GRD and do not feel socially judged and/or disapproved had the best scores to total EC and its parameters. Those who feel they cannot eat meals with their colleagues, friends and/or family at meetings or events presented lower EC scores. Individuals with GRDs usually face social difficulties because of GFD for their entire lives. For example, in the Brazilian study, participants mentioned that when invited to social events, they took their food to avoid gluten-containing foods if they planned to participate. Most reported that they preferred not to participate due to dietary restrictions [71]. In our study, participants who answered that “Always/almost always” felt socially judged for having GRDs had lower EC scores than those who reported that “never/almost never”. This association also occurred for the feeling that they cannot live a normal life because of GRDs, since individuals who reported that “Always/almost always”, had lower EC scores than those who reported that “never/almost never” feel it. People who cannot feel a normal life with GRD and feel socially disapproved because of GFD may face lower emotional well-being and perception of food insecurity [76], which could explain our findings.

Our study showed that among individuals with GRDs, EC is directly associated with schooling, income, cooking skills, following a non-conforming GFD, and having a good food acceptance. These results are in line with previous studies where EC was associated with socioeconomic data [15,26,36,40,42,44,77,78,79,80]. Another important result in this study was the overall percentage of competent eaters (56.1%), showing that people with some GRDs tend to be more concerned about their diet.

The strengths of this research were the sample size (*n* = 1030) from all over the country, since it deals with individuals with specific conditions such as GRD. Another strength was using an instrument previously validated for the Brazilian population [45,51]. One of the inclusion criteria was “having at least one gluten-related disorder diagnosed by a physician” however, proof of diagnosis was not required, generating a potential risk of bias.

## 5. Conclusions

Adherence to a GFD entails several difficulties, and, in this study, the high cost of GF products was the main reason for intentionally breaking the GFD. Preparing GF food at home is the main contributor to following a GFD and is associated with high EC scores. GFD adherence and culinary practices were associated with an increased EC total score. Greater dissatisfaction with GF products was associated with lower EC scores feeling socially judged. In general, EC seemed to be associated with positive eating behaviors and a good acceptance of dietary changes. GRD individuals showed to be more competent eaters than the general Brazilian population. Our findings support the need for greater attention to the development of EC in individuals with GRDs through strategies and ways of nutrition education, policy, and social and individual intervention allowing them to lead easily with the GRD and its treatment.

## Figures and Tables

**Table 1:** Crossing the scores of the ecSI2.0™BR and sociodemographic data (*n* = 1030).

	Eating Attitude	Food Acceptance	Internal Regulation	Contextual Skills	Total	ecSI2.0™BR ≥ 32
	Mean (SD)	Mean (SD)	Mean (SD)	Mean (SD)	Mean (SD)	Freq. (%)
Gender *						
Female (*n* = 986)	11.73 (3.69) ^a^	5.01 (2.24) ^a^	4.06 (1.43) ^a^	11.08 (3.10) ^a^	31.88 (7.97) ^a^	554 (56.2%) ^a^
Male (*n* = 44)	12.18 (3.87) ^a^	4.91 (1.99) ^a^	4.45 (1.23) ^a^	11.07 (3.03) ^a^	32.61 (7.70) ^a^	24 (54.5%) ^a^
*p*	0.432 *	0.771 *	0.071 *	0.980 *	0.550 *	0.830 ***
Age *						
Up to 40 years old (*n* = 499)	11.61 (3.73) ^a^	4.87 (2.26) ^a^	4.00 (1.45) ^a^	10.59 (3.18) ^a^	31.07 (8.12) ^a^	255 (51.1%) ^a^
40 years or older (*n* = 531)	11.89 (3.67) ^a^	5.13 (2.20) ^a^	4.14 (1.40) ^a^	11.54 (2.94) ^b^	32.70 (7.72) ^b^	323 (60.8%) ^b^
*p*	0.216 *	0.060 *	0.113 *	0.000 *	0.001 *	0.002 ***
Educational level **						
Up to high school (*n* = 158)	11.35 (3.86) ^a^	4.83 (2.23) ^a^	4.18 (1.49) ^a^	11.14 (3.10) ^a^	31.50 (7.88) ^a^	81 (51.3%) ^a^
Undergraduate (*n* = 407)	11.71 (3.68) ^a^	4.85 (2.24) ^a^	4.05 (1.48) ^a^	10.82 (3.22) ^a^	31.42 (8.11) ^a^	221 (54.3%) ^a^
Graduate (*n* = 465)	11.93 (3.65) ^a^	5.20 (2.21) ^b^	4.06 (1.35) ^a^	11.29 (2.96) ^a^	32.49 (7.82) ^a^	276 (59.4%) ^a^
*p*	0.223 **	0.034 **	0.574 **	0.075 **	0.109 **	0.133 ***
Income **						
Up to R$3000.00 (*n* = 254)	11.20 (3.83) ^a^	4.72 (2.23) ^a^	3.98 (1.51) ^a^	10.67 (3.21) ^a^	30.57 (8.16) ^a^	125 (49.2%) ^a^
R$3001.00 to R$5000.00 (*n* = 201)	11.67 (3.67) ^a^	4.84 (2.26) ^ab^	4.09 (1.45) ^a^	10.95 (2.98) ^a^	31.54 (7.80) ^ab^	106 (52.7%) ^ab^
R$5001.00 to R$10,000.00 (*n* = 252)	12.04 (3.69) ^a^	5.26 (2.12) ^b^	4.14 (1.35) ^a^	11.31 (3.20) ^a^	32.75 (7.94) ^b^	156 (61.9%) ^b^
R$10,001.00 to R$20,000.00 (*n* = 192)	11.91 (3.58) ^a^	5.06 (2.23) ^ab^	4.08 (1.43) ^a^	11.19 (2.90) ^a^	32.24 (7.64) ^ab^	114 (59.4%) ^ab^
More than R$20,000.00 (*n* = 57)	11.88 (3.16) ^a^	5.44 (2.65) ^b^	3.98 (1.29) ^a^	11.02 (3.31) ^a^	32.32 (8.30) ^ab^	33 (57.9%) ^ab^
*p*	0.107 **	0.029 **	0.766 **	0.196 **	0.031 **	0.038 ***
**Do you have any other restrictions besides gluten?**						
Yes (*n* = 669)	11.66 (3.78) ^a^	5.09 (2.23) ^a^	4.08 (1.41) ^a^	11.26 (3.00) ^b^	32.09 (7.97) ^a^	381 (57.0%) ^a^
No (*n* = 361)	11.93 (3.55) ^a^	4.84 (2.23) ^a^	4.07 (1.45) ^a^	10.74 (3.23) ^a^	31.58 (7.93) ^a^	197 (54.6%) ^a^
*p*	0.271 *	0.091 *	0.964 *	0.011 *	0.328 *	0.463 ***
**Do you follow a gluten-free diet?**						
Never/almost never (*n* = 7)	11.00 (2.77) ^ab^	3.57 (1.90) ^a^	3.86 (1.77) ^ab^	8.14 (5.08) ^a^	26.57 (7.50) ^b^	2 (28.6%) ^ab^
Sometimes (*n* = 41)	10.10 (4.04) ^a^	4.46 (2.47) ^a^	3.46 (1.43) ^a^	8.37 (3.20) ^a^	26.39 (8.31) ^a^	11 (26.8%) ^a^
Always/almost always (*n* = 982)	11.83 (3.68) ^b^	5.04 (2.22) ^a^	4.10 (1.42) ^b^	11.21 (3.01) ^b^	32.18 (7.85) ^b^	565 (57.5%) ^b^
*p*	0.012 **	0.063 **	0.018 **	0.000 **	0.000 **	0.000 ***
**How often do you buy gluten-free products?**						
Never/almost never (*n* = 38)	11.16 (4.12) ^a^	5.47 (2.42) ^a^	3.89 (1.54) ^a^	10.71 (4.15) ^a^	31.24 (9.04) ^a^	21 (55.3%) ^a^
Sometimes (*n* = 232)	11.45 (3.81) ^a^	4.98 (2.12) ^a^	3.98 (1.41) ^a^	10.96 (3.23) ^a^	31.38 (8.05) ^a^	121 (52.2%) ^a^
Always/almost always (*n* = 760)	11.87 (3.64) ^a^	4.99 (2.26) ^a^	4.11 (1.42) ^a^	11.13 (2.99) ^a^	32.11 (7.87) ^a^	436 (57.4%) ^a^
*p*	0.190 **	0.419 **	0.331 **	0.572 **	0.407**	0.373 ***
**Are you satisfied with the gluten-free products you buy?**						
Not satisfied (*n* = 67)	9.97 (4.17) ^a^	4.36 (2.44) ^a^	3.52 (1.72) ^a^	9.31 (3.92) ^a^	27.16 (9.28) ^a^	23 (34.3%) ^a^
Little satisfied (*n* = 452)	11.02 (3.56) ^a^	4.86 (2.15) ^a^	4.01 (1.36) ^b^	10.65 (3.00) ^b^	30.53 (7.53) ^b^	218 (48.2%) ^a^
Satisfied (*n* = 497)	12.56 (3.54) ^b^	5.25 (2.25) ^b^	4.20 (1.41) ^b^	11.71 (2.85) ^c^	33.81 (7.62) ^c^	328 (66.0%) ^b^
*p*	0.000 **	0.000 **	0.000 **	0.000 **	0.000 **	0.000 ***
**How often do you consume gluten-free products prepared at home?**						
Never/almost never (*n* = 44)	9.68 (3.70) ^a^	4.45 (2.28) ^ab^	3.73 (1.73) ^ab^	9.55 (4.58) ^a^	27.41 (8.75) ^a^	15 (34.1%) ^a^
Sometimes (*n* = 228)	11.27 (3.74) ^b^	4.66 (2.10) ^a^	3.89 (1.48) ^a^	10.32 (3.17) ^a^	30.14 (8.13) ^a^	107 (46.9%) ^a^
Always/almost always (*n* = 758)	12.02 (3.64) ^ac^	5.14 (2.26) ^b^	4.15 (1.38) ^b^	11.4 (2.89) ^b^	32.7 (7.69) ^b^	456 (60.2%) ^b^
*p*	0.000 **	0.004 **	0.012 **	0.000 **	0.000 **	0.000 ***
**If you consume gluten-free products prepared at home, who prepares them?**						
Myself (*n* = 902)	11.94 (3.65) ^b^	5.09 (2.20) ^b^	4.12 (1.41) ^b^	11.31 (2.97) ^b^	32.46 (7.77) ^b^	531 (58.9%) ^b^
Another person (*n* = 62)	10.74 (3.90) ^a^	4.11 (2.28) ^a^	3.35 (1.48) ^a^	9.58 (3.45) ^a^	27.79 (8.29) ^a^	23 (37.1%) ^a^
*p*	0.013 **	0.001 **	0.000 *	0.000 *	0.000 *	0.000 ***
**Do the people you live with help you to have a gluten-free diet?**						
Never/almost never (*n* = 85)	9.87 (4.14) ^a^	4.38 (2.42) ^a^	3.58 (1.59) ^a^	9.67 (3.89) ^a^	27.49 (9.04) ^a^	32 (37.6%) ^a^
Sometimes (*n* = 216)	10.34 (3.95) ^a^	4.56 (2.13) ^a^	3.84 (1.41) ^a^	10.16 (3.13) ^a^	28.90 (8.20) ^a^	82 (38.0%) ^a^
Always/almost always (*n* = 729)	12.39 (3.37) ^b^	5.21 (2.21) ^b^	4.20 (1.39) ^b^	11.52 (2.86) ^b^	33.32 (7.29) ^b^	464 (63.6%) ^b^
*p*	0.000 **	0.000 **	0.000 **	0.000 **	0.000 **	0.000 ***
**I feel socially judged and/or disapproved of for having this gluten-related disorder.**						
Never/almost never (*n* = 208)	13.15 (3.56) ^c^	5.52 (2.14) ^c^	4.51 (1.32) ^b^	11.99 (2.73) ^c^	35.17 (7.16) ^c^	146 (70.2%) ^c^
Sometimes (*n* = 494)	11.96 (3.44) ^b^	5.07 (2.16) ^b^	4.06 (1.38) ^b^	11.17 (3.08) ^b^	32.26 (7.65) ^b^	289 (58.5%) ^b^
Always/almost always (*n* = 328)	10.56 (3.80) ^a^	4.57 (2.31) ^a^	3.83 (1.50) ^a^	10.37 (3.17) ^a^	29.32 (8.04) ^a^	143 (43.6%) ^a^
*p*	0.000 **	0.000 **	0.000 **	0.000 **	0.000 **	0.000 ***
**I feel I can’t live a normal life because of this gluten-related disorder.**						
Never/almost never (*n* = 262)	13.26 (3.36) ^c^	5.63 (2.24) ^c^	4.39 (1.35) ^b^	12.00 (2.80) ^c^	35.28 (7.14) ^c^	192 (73.3%) ^a^
Sometimes (*n* = 475)	11.71 (3.51) ^b^	5.03 (2.13) ^b^	4.00 (1.42) ^a^	11.11 (3.04) ^b^	31.85 (7.68) ^b^	268 (56.4%) ^b^
Always/almost always (*n* = 293)	10.47 (3.80) ^a^	4.40 (2.23) ^a^	3.91 (1.46) ^a^	10.21 (3.19) ^a^	29.00 (7.94) ^a^	118 (40.3%) ^c^
*p*	0.000 **	0.000 **	0.000 **	0.000 **	0.000 **	0.000 ***
**How often do you feel that you cannot eat meals with your colleagues, friends and/or family at meetings or events?**						
Never/almost never (*n* = 99)	13.14 (3.51) ^b^	5.48 (2.24) ^b^	4.47 (1.34) ^b^	11.97 (2.95) ^b^	35.07 (7.60) ^b^	68 (68.7%) ^b^
Sometimes (*n* = 321)	12.42 (3.55) ^b^	5.24 (2.19) ^b^	4.12 (1.41) ^ab^	11.39 (2.97) ^b^	33.17 (7.73) ^b^	195 (60.7%) ^b^
Always/almost always (*n* = 610)	11.18 (3.69) ^a^	4.80 (2.23) ^a^	3.98 (1.44) ^a^	10.77 (3.14) ^a^	30.73 (7.90) ^a^	315 (51.6%) ^a^
*p*	0.000 **	0.001 **	0.005 **	0.000 **	0.000 **	0.001 ***

* Student *t*-test. ** ANOVA with Tukey post-hoc test. *** Pearson’s chi-squared test. For each variable, the same letters (a, b, c) comparing lines do not differ significantly.

## Data Availability

Data sharing is not applicable to this article.

## Supplementary Materials

The following are available online at https://www.mdpi.com/article/10.3390/nu14142815/s1. Table S1. Factors associated with a gluten-free diet (*n* = 1030). Table S2. Crossing the scores of the ecSI2.0™BR and sociodemographic data of celiac patients (*n* = 781). Table S3. Crossing the scores of the ecSI2.0™BR and sociodemographic data of non-celiac patients (*n* = 249).
