# Supplementary material for: Eating Competence and Aspects Related to a Gluten-Free Diet in Brazilian Adults with Gluten-Related Disorders
Source: Nutrients. 2022 Jul 8;14(14):2815. doi: 10.3390/nu14142815 (PMC9319171; doi:10.3390/nu14142815)
Supplement: Supplementary file 1 [file nutrients-14-02815-s001.zip › nutrients-1795642-supplementary.pdf]

Table S1. Factors associated with the gluten-free diet.

	Freq.*	%
**What is the main reason you follow a gluten-free diet?**		
Gluten/wheat allergy	95	7.9%
Celiac disease	781	64.9%
Dermatitis herpetiformis	84	7.0%
Non-celiac gluten sensitivity	244	20.2%
**If you buy sometimes or never buy special gluten-free products (e.g., gluten-free bread, gluten-free pasta, gluten-free cake, etc.), why?**		
They are expensive	206	36.9%
I don't like	51	9.1%
Stores are difficult to access	79	14.1%
I can't find gluten-free products to buy	44	7.9%
I don't know this type of product	0	0%
I don't trust these products	16	2.9%
I prepare my gluten-free products at home	136	24.3%
Other	27	4.8%
**If you do not consume special gluten-free products (e.g., gluten-free bread, gluten-free cake, gluten-free cookies, etc.) prepared at home, why?**		
Ingredients are expensive	257	38.6%
I can't easily find the ingredients to prepare these recipes	138	20.7%
I don't know/I don't like to cook, OR I don't have someone to prepare my food	79	11.9%
I don't have time to cook	141	21.2%
Other	51	7.6%
**What helps you maintain your gluten-free diet?**		
I prepare gluten-free foods at home.	852	46.9%
Someone prepares gluten-free food for me	256	14.1%
I have easy access to this type of product (restaurants, stores, markets, fairs, people, internet, etc.)	259	14.2%
I have the financial availability to purchase gluten-free products	328	18.0%
Other	123	6.8%
**What are the difficulties you have in following a gluten-free diet?**		
I don't understand what foods I can and can't eat	23	1.2%
I don't have time to prepare different meals	179	9.0%
Gluten-free food tastes unpleasant	117	5.9%
Gluten-free foods have an unpleasant texture	173	8.8%
Gluten-free foods are expensive	583	29.5%
The health professional does not provide enough guidance to follow a gluten-free diet	140	7.1%
I feel different from others following the gluten-free diet	219	11.1%
I don't understand the food label	30	1.5%
Other	135	6.8%
I have no difficulty	377	19.1%

* The frequencies can exceed 1030 since it allows multiple responses.

Table S2. Crossing the scores of the ecSI2.0™BR and sociodemographic data of celiac patients (*n* = 781).

	Eating attitude	Food acceptance	Internal regulation	Contextual skills	Total	ecSI2.0™BR ⩾ 32
	**Mean (SD)**	**Mean (SD)**	**Mean (SD)**	**Mean (SD)**	**Mean (SD)**	**Freq. (%)**
**Gender**						
Female (n=742)	11.95 (3.56)[a]	5.00 (2.26)[a]	4.08 (1.44)[a]	11.20 (3.06)[a]	32.22 (7.92)[a]	433 (58.4%)[a]
Male (n=39)	12.15 (3.89)[a]	4.77 (1.98)[a]	4.36 (1.20)[a]	10.95 (3.09)[a]	32.23 (7.74)[a]	21.00 (53.8%)[a]
p	0.729*	0.539*	0.231*	0.624*	0.993*	0.578***
**Age**						
Up to 40 years old (n=396)	11.80 (3.53)[a]	4.85 (2.29)[a]	3.99 (1.44)[a]	10.70 (3.16)[a]	31.34 (7.95)[a]	211 (53.3%)[a]
40 years or older (n=385)	12.13 (3.62)[a]	5.12 (2.19)[a]	4.19 (1.41)[a]	11.68 (2.87)[b]	33.12 (7.76)[b]	243 (63.1%)[b]
p	0.199*	0.086*	0.053*	0.000*	0.002*	0.005***
**Educational level**						
Up to high school (n=114)	11.75 (3.77)[a]	4.76 (2.24)[a]	4.26 (1.49)[a]	11.40 (3.08)[a]	32.18 (7.81)[a]	61 (53.5%)[a]
Undergraduate (n=306)	11.95 (3.52)[a]	4.90 (2.22)[a]	4.07 (1.45)[a]	10.99 (3.22)[a]	31.91 (7.96)[a]	175 (57.2%)[a]
Graduate (n=361)	12.04 (3.57)[a]	5.12 (2.26)[a]	4.06 (1.39)[a]	11.28 (2.91)[a]	32.50 (7.89)[a]	218 (60.4%)[a]
p	0.746**	0.231**	0.382**	0.332**	0.623**	0.393***
**Income**						
Up to R$ 3,000.00 (n=185)	11.63 (3.72)[a]	4.71 (2.27)[a]	4.05 (1.51)[a]	10.82 (3.35)[a]	31.21 (8.29)[a]	98 (53.0%)[a]
R$ 3,001.00 to R$ 5,000.00 (n=159)	11.77 (3.52)[a]	4.82 (2.30)[a]	4.09 (1.48)[a]	10.93 (3.00)[a]	31.62 (7.89)[a]	85 (53.5%)[a]
R$ 5,001.00 to R$ 10,000.00 (n=196)	12.35 (3.47)[a]	5.22 (2.11)[a]	4.23 (1.31)[a]	11.61 (3.00)[a]	33.41 (7.53)[a]	127 (64.8%)[a]
R$ 10,001.00 to R$20,000.00 (n=147)	12.01 (3.45)[a]	5.06 (2.28)[a]	4.03 (1.40)[a]	11.30 (2.75)[a]	32.40 (7.42)[a]	88 (59.9%)[a]
More than R$ 20,000.00 (n=39)	12.03 (3.41)[a]	5.13 (2.74)[a]	3.85 (1.44)[a]	10.74 (3.48)[a]	31.74 (9.16)[a]	24 (61.5%)[a]
p	0.340**	0.212**	0.486**	0.082**	0.073**	0.117***
**Do you have any other restrictions besides gluten?**						
Yes (n=484)	11.87 (3.71)[a]	5.08 (2.25)[a]	4.06 (1.44)[a]	11.33 (2.95)[a]	32.34 (8.01)[a]	286 (59.1%)[a]
No (n=297)	12.10 (3.36)[a]	4.83 (2.23)[a]	4.15 (1.40)[a]	10.94 (3.22)[a]	32.02 (7.73)[a]	168 (56.6%)[a]
p	0.390*	0.136*	0.363*	0.081*	0.586*	0.487***
**Do you follow a gluten-free diet?**						
Never/almost never (n=4)	11.75 (2.22)[a]	3.50 (2.65)[a]	4.75 (1.50)[a]	8.25 (6.24)[ab]	28.25 (8.54)[ab]	1 (25.0%)[ab]
Sometimes (n=11)	10.64 (4.92)[a]	4.18 (3.06)[a]	3.64 (1.86)[a]	7.00 (2.97)[a]	25.45 (8.82)[b]	3 (27.3%)[a]
Always/almost always (n=766)	11.98 (3.56)[a]	5.00 (2.23)[a]	4.10 (1.42)[a]	11.26 (3.00)[b]	32.34 (7.85)[a]	450 (58.7%)[b]
p	0.463**	0.201**	0.373**	0.000**	0.010**	0.044***
**How often do you buy gluten-free products?**						
Never/almost never (n=23)	12.52 (3.82)[a]	6.22 (2.15)[ab]	4.30 (1.49)[a]	11.78 (4.19)[a]	34.83 (8.17)[a]	17 (73.9%)[a]
Sometimes (n=154)	11.88 (3.77)[a]	5.01 (2.24)[a]	3.98 (1.47)[a]	11.32 (3.15)[a]	32.19 (8.23)[a]	89 (57.8%)[a]
Always/almost always (n=604)	11.96 (3.52)[a]	4.93 (2.24)[a]	4.11 (1.41)[a]	11.13 (2.99)[a]	32.13 (7.81)[a]	348 (57.6%)[a]
p	0.727**	0.026**	0.455**	0.498**	0.275**	0.297***
**Are you satisfied with the gluten-free products you buy?**						
Not satisfied (n=48)	10.06 (4.09)[a]	4.33 (2.63)[a]	3.42 (1.72)[a]	9.69 (3.84)[a]	27.50 (9.15)[a]	18 (37.5%)[a]
Little satisfied (n=333)	11.26 (3.41)[b]	4.83 (2.10)[ab]	4.02 (1.40)[b]	10.76 (2.93)[b]	30.86 (7.34)[b]	169 (50.8%)[a]
Satisfied (n=391)	12.80 (3.45)[c]	5.21 (2.30)[b]	4.23 (1.38)[b]	11.75 (2.90)[c]	33.99 (7.76)[c]	262 (67.0%)[b]
p	0.000**	0.008**	0.000**	0.000**	0.000**	0.000***
**How often do you consume gluten-free products prepared at home?**						
Never/almost never (n=33)	9.85 (3.48)[a]	3.97 (2.13)[a]	3.58 (1.89)[a]	9.64 (4.68)[a]	27.03 (8.68)[a]	12 (36.4%)[a]
Sometimes (n=159)	11.53 (3.75)[b]	4.60 (2.06)[ab]	3.86 (1.52)[ab]	10.55 (3.12)[ab]	30.53 (8.15)[b]	79 (49.7%)[a]
Always/almost always (n=589)	12.20 (3.49)[b]	5.15 (2.27)[b]	4.18 (1.36)[b]	11.44 (2.88)[b]	32.97 (7.62)[b]	363 (61.6%)[b]
p	0.000**	0.001**	0.004**	0.000**	0.000**	0.001***
**If you consume gluten-free products prepared at home, who prepares them?**						
Myself (n=686)	12.16 (3.51)[b]	5.08 (2.20)[b]	4.15 (1.40)[b]	11.4 (2.93)[b]	32.78 (7.68)[b]	416 (60.6%)[b]
Another person (n=49)	10.96 (3.85)[a]	4.00 (2.38)[a]	3.29 (1.55)[a]	9.80 (3.55)[a]	28.04 (8.56)[a]	20 (40.8%)[a]
p	0.022*	0.001*	0.000*	0.000*	0.000*	0.006***
**Do the people you live with help you to have a gluten-free diet?**						
Never/almost never (n=52)	10.31 (4.26)[a]	4.33 (2.41)[a]	3.63 (1.66)[a]	9.87 (4.13)[a]	28.13 (9.20)[a]	22 (42.3%)[a]
Sometimes (n=137)	10.66 (3.88)[a]	4.55 (2.18)[ab]	3.78 (1.48)[ab]	10.09 (3.11)[a]	29.09 (8.41)[a]	57 (41.6%)[a]
Always/almost always (n=592)	12.41 (3.32)[b]	5.14 (2.22)[b]	4.20 (1.38)[b]	11.55 (2.85)[b]	33.30 (7.34)[b]	375 (63.3%)[b]

p	0.000**	0.002**	0.000**	0.000**	0.000**	0.000***
**I feel socially judged and/or disapproved of for having this gluten-related disorder.**						
Never/almost never (n=146)	13.61 (3.33)^{c}	5.55 (2.09)^{b}	4.62 (1.29)^{b}	12.10 (2.77)^{c}	35.87 (7.04)^{c}	110 (75.3%)^{c}
Sometimes (n=376)	12.10 (3.32)^{b}	5.10 (2.16)^{b}	4.05 (1.38)^{a}	11.33 (2.99)^{b}	32.57 (7.47)^{b}	226 (60.1%)^{b}
Always/almost always (n=259)	10.83 (3.69)^{a}	4.50 (2.36)^{a}	3.86 (1.50)^{a}	10.46 (3.16)^{a}	29.65 (8.09)^{a}	118 (45.6%)^{a}
p	0.000**	0.000**	0.000**	0.000**	0.000**	0.000***
**I feel I can't live a normal life because of this gluten-related disorder.**						
Never/almost never (n=182)	13.48 (3.29)^{c}	5.57 (2.22)^{c}	4.39 (1.35)^{b}	11.97 (2.90)^{c}	35.41 (7.36)^{b}	137 (75.3%)^{c}
Sometimes (n=371)	11.96 (3.34)^{b}	5.02 (2.14)^{b}	4.02 (1.41)^{a}	11.26 (2.94)^{b}	32.26 (7.54)^{b}	217 (58.5%)^{b}
Always/almost always (n=228)	10.75 (3.73)^{a}	4.46 (2.31)^{a}	3.98 (1.48)^{a}	10.43 (3.21)^{a}	29.61 (7.98)^{a}	100 (43.9%)^{a}
p	0.000**	0.000**	0.005**	0.000**	0.000**	0.000***
**How often do you feel that you cannot eat meals with your colleagues, friends and/or family at meetings or events?**						
Never/almost never (n=65)	13.72 (3.36)^{c}	5.32 (2.23)^{ab}	4.68 (1.16)^{b}	12.22 (2.63)^{b}	35.94 (7.04)^{c}	46 (70.8%)^{b}
Sometimes (n=233)	12.68 (3.37)^{b}	5.26 (2.21)^{b}	4.15 (1.43)^{a}	11.53 (3.00)^{ab}	33.63 (7.74)^{b}	148 (63.5%)^{ab}
Always/almost always (n=483)	11.37 (3.57)^{a}	4.81 (2.25)^{a}	3.98 (1.44)^{a}	10.88 (3.10)^{a}	31.04 (7.84)^{a}	260 (53.8%)^{a}
p	0.000**	0.017**	0.001**	0.000**	0.000**	0.005***

* Student t-test ** Anova with Tukey post-hoc test. *** Pearson's chi-squared test. **** Fisher's exact test. For each variable, the same letters comparing lines do not differ significantly.

Table S3. Crossing the scores of the ecSI2.0™BR and sociodemographic data of non-celiac patients (*n* = 249).

	Eating attitude	Food acceptance	Internal regulation	Contextual skills	Total	ecSI2.0™BR $\geqslant$ 32
	Mean (SD)	Mean (SD)	Mean (SD)	Mean (SD)	Mean (SD)	Freq. (%)
Gender						
Female (n=244)	11.07 (4.00)^{a}	5.05 (2.20)^{a}	4.00 (1.42)^{a}	10.73 (3.19)^{a}	30.85 (8.05)^{a}	121 (49.6%)^{a}
Male (n=5)	12.40 (4.16)^{a}	6.00 (1.87)^{a}	5.20 (1.30)^{a}	12.00 (2.65)^{a}	35.60 (7.47)^{a}	3 (60.0%)^{a}
p	0.464*	0.339*	0.062*	0.377*	0.192*	0.684****
Age						
Up to 40 years old (n=103)	10.86 (4.33)^{a}	4.95 (2.18)^{a}	4.03 (1.52)^{a}	10.17 (3.20)^{a}	30.02 (8.70)^{a}	44 (42.7%)^{a}
40 years or older (n=146)	11.27 (3.75)^{a}	5.15 (2.21)^{a}	4.01 (1.36)^{a}	11.16 (3.11)^{a}	31.60 (7.53)^{a}	80 (54.8%)^{a}
p	0.446*	0.482*	0.933*	0.015*	0.128*	0.061***
Educational level						
Up to high school (n=44)	10.32 (3.96)^{a}	5.00 (2.21)^{a}	3.98 (1.50)^{a}	10.45 (3.08)^{a}	29.75 (7.86)^{ab}	20 (45.5%)^{a}
Undergraduate (n=101)	10.98 (4.07)^{a}	4.67 (2.29)^{a}	3.98 (1.57)^{a}	10.30 (3.19)^{a}	29.93 (8.39)^{a}	46 (45.5%)^{a}
Graduate (n=104)	11.55 (3.92)^{a}	5.48 (2.03)^{a}	4.08 (1.24)^{a}	11.33 (3.15)^{a}	32.43 (7.61)^{b}	58 (55.8%)^{a}
p	0.215**	0.030**	0.869**	0.053**	0.046**	0.280***
Income						
Up to R$ 3.000.00 (n=69)	10.06 (3.91)^{a}	4.74 (2.14)^{a}	3.80 (1.49)^{a}	10.28 (2.78)^{a}	28.87 (7.62)^{a}	27 (39.1%)^{a}
R$ 3.001.00 to R$ 5.000.00 (n=42)	11.26 (4.21)^{a}	4.88 (2.12)^{a}	4.10 (1.34)^{a}	11.02 (2.97)^{a}	31.26 (7.53)^{a}	21 (50.0%)^{a}
R$ 5.001.00 to R$ 10.000.00 (n=56)	10.95 (4.23)^{a}	5.41 (2.20)^{a}	3.82 (1.45)^{a}	10.27 (3.65)^{a}	30.45 (8.94)^{a}	29 (51.8%)^{a}
R$ 10.001.00 to R$20.000.00 (n=45)	11.58 (4.00)^{a}	5.04 (2.09)^{a}	4.24 (1.52)^{a}	10.84 (3.36)^{a}	31.71 (8.41)^{a}	26 (57.8%)^{a}
More than R$ 20.000.00 (n=18)	11.56 (2.62)^{a}	6.11 (2.40)^{a}	4.28 (0.83)^{a}	11.61 (2.91)^{a}	33.56 (6.07)^{a}	9 (50.0%)^{a}
p	0.269**	0.117**	0.350**	0.386**	0.148**	0.371***
Do you have any other restrictions besides gluten?						
Yes (n=185)	11.10 (3.90)^{a}	5.12 (2.18)^{a}	4.13 (1.34)^{a}	11.08 (3.13)^{b}	31.43 (7.83)^{a}	95 (51.4%)^{a}
No (n=64)	11.11 (4.30)^{a}	4.91 (2.26)^{a}	3.70 (1.62)^{a}	9.81 (3.15)^{a}	29.53 (8.57)^{a}	29 (45.3%)^{a}
p	0.983*	0.495*	0.061*	0.006*	0.104*	0.405***
Do you follow a gluten-free diet?						
Never/almost never (n=3)	10.00 (3.61)^{a}	3.67 (0.58)^{a}	2.67 (1.53)^{ab}	8.00 (4.36)^{ab}	24.33 (6.81)^{ab}	1 (33.3%)^{ab}
Sometimes (n=30)	9.90 (3.74)^{a}	4.57 (2.27)^{a}	3.40 (1.28)^{a}	8.87 (3.18)^{a}	26.73 (8.25)^{a}	8 (26.7%)^{a}
Always/almost always (n=216)	11.28 (4.02)^{a}	5.16 (2.19)^{a}	4.13 (1.42)^{b}	11.06 (3.07)^{b}	31.62 (7.85)^{b}	115 (53.2%)^{b}
p	0.185**	0.208**	0.008**	0.001**	0.003**	0.021***

How often do you buy gluten-free products?						
Never/almost never (n=15)	9.07 (3.75)^{a}	4.33 (2.44)^{a}	3.27 (1.44)^{a}	9.07 (3.63)^{a}	25.73 (7.59)^{a}	4 (26.7%)^{a}
Sometimes (n=78)	10.60 (3.77)^{ab}	4.94 (1.87)^{a}	3.97 (1.31)^{a}	10.26 (3.29)^{ab}	29.77 (7.49)^{ab}	32 (41.0%)^{ab}
Always/almost always (n=156)	11.54 (4.06)^{b}	5.21 (2.31)^{a}	4.12 (1.47)^{a}	11.17 (3.00)^{b}	32.03 (8.13)^{b}	88 (56.4%)^{b}
p	0.029**	0.278**	0.083**	0.012**	0.004**	0.015***
Are you satisfied with the gluten-free products you buy?						
Not satisfied (n=19)	9.74 (4.48)^{a}	4.42 (1.92)^{a}	3.79 (1.72)^{a}	8.37 (4.09)^{a}	26.32 (9.79)^{a}	5 (26.3%)^{a}
Little satisfied (n=119)	10.34 (3.88)^{ab}	4.94 (2.30)^{a}	3.97 (1.25)^{a}	10.34 (3.18)^{b}	29.60 (7.97)^{ab}	49 (41.2%)^{ab}
Satisfied (n=106)	12.11 (3.81)^{b}	5.40 (2.05)^{a}	4.08 (1.53)^{a}	11.58 (2.67)^{b}	33.17 (7.07)^{b}	66 (62.3%)^{b}
p	0.001**	0.109**	0.660**	0.000**	0.000**	0.001***
How often do you consume gluten-free products prepared at home?						
Never/almost never (n=11)	9.18 (4.42)^{a}	5.91 (2.17)^{a}	4.18 (1.08)^{a}	9.27 (4.47)^{ab}	28.55 (9.27)^{ab}	3 (27.3%)^{ab}
Sometimes (n=69)	10.68 (3.68)^{a}	4.81 (2.18)^{a}	3.94 (1.37)^{a}	9.81 (3.26)^{a}	29.25 (8.07)^{a}	28 (40.6%)^{a}
Always/almost always (n=169)	11.40 (4.07)^{a}	5.12 (2.20)^{a}	4.04 (1.47)^{a}	11.24 (2.95)^{b}	31.79 (7.87)^{b}	93 (55.0%)^{b}
p	0.121**	0.268**	0.826**	0.002**	0.050**	0.040***
If you consume gluten-free products prepared at home. who prepares them?						
Myself (n=216)	11.26 (4.01)^{a}	5.09 (2.19)^{a}	4.05 (1.43)^{a}	11.04 (3.08)^{b}	31.45 (8.01)^{b}	115 (53.2%)^{b}
Another person (n=13)	9.92 (4.11)^{a}	4.54 (1.90)^{a}	3.62 (1.19)^{a}	8.77 (3.03)^{a}	26.85 (7.40)^{a}	3 (23.1%)^{a}
p	0.243*	0.373*	0.284*	0.010*	0.045*	0.045****
Do the people you live with help you to have a gluten-free diet?						
Never/almost never (n=33)	9.18 (3.90)^{a}	4.45 (2.48)^{a}	3.48 (1.50)^{a}	9.36 (3.53)^{a}	26.48 (8.81)^{a}	10 (30.3%)^{a}
Sometimes (n=79)	9.78 (4.03)^{a}	4.56 (2.06)^{a}	3.94 (1.27)^{ab}	10.29 (3.18)^{ab}	28.57 (7.88)^{a}	25 (31.6%)^{a}
Always/almost always (n=137)	12.32 (3.58)^{b}	5.51 (2.12)^{b}	4.20 (1.46)^{b}	11.36 (2.95)^{b}	33.39 (7.09)^{b}	89 (65.0%)^{b}
p	0.000**	0.002**	0.029**	0.001**	0.000**	0.000***
I feel socially judged and/or disapproved of for having this gluten-related disorder.						
Never/almost never (n=62)	12.06 (3.87)^{b}	5.47 (2.29)^{a}	4.26 (1.35)^{b}	11.74 (2.63)^{b}	33.53 (7.22)^{b}	36 (58.1%)^{b}
Sometimes (n=118)	11.51 (3.80)^{b}	4.99 (2.19)^{a}	4.09 (1.40)^{ab}	10.67 (3.31)^{ab}	31.26 (8.14)^{b}	63 (53.4%)^{ab}
Always/almost always (n=69)	9.54 (4.05)^{a}	4.84 (2.11)^{a}	3.68 (1.49)^{a}	10.01 (3.21)^{a}	28.07 (7.81)^{a}	25 (36.2%)^{a}
p	0.000**	0.231**	0.050**	0.007**	0.000**	0.025***
I feel I can't live a normal life because of this gluten-related disorder.						
Never/almost never (n=80)	12.75 (3.47)^{b}	5.79 (2.29)^{b}	4.39 (1.35)^{b}	12.08 (2.55)^{b}	35.00 (6.64)^{c}	55 (68.8%)^{c}
Sometimes (n=104)	10.85 (3.97)^{a}	5.05 (2.09)^{b}	3.94 (1.46)^{ab}	10.55 (3.34)^{a}	30.38 (8.00)^{b}	51 (49.0%)^{b}
Always/almost always (n=65)	9.48 (3.93)^{a}	4.22 (1.96)^{a}	3.69 (1.39)^{a}	9.46 (3.03)^{a}	26.85 (7.45)^{a}	18 (27.7%)^{a}
p	0.000**	0.000**	0.010**	0.000**	0.000**	0.000***
How often do you feel that you cannot eat meals with your colleagues. friends and/or family at meetings or events?						
Never/almost never (n=34)	12.03 (3.57)^{ab}	5.79 (2.25)^{b}	4.09 (1.58)^{a}	11.50 (3.48)^{a}	33.41 (8.42)^{b}	22 (64.7%)^{a}
Sometimes (n=88)	11.73 (3.91)^{b}	5.18 (2.16)^{ab}	4.05 (1.36)^{a}	11.02 (2.87)^{a}	31.98 (7.60)^{ab}	47 (53.4%)^{a}
Always/almost always (n=127)	10.42 (4.07)^{a}	4.80 (2.17)^{a}	3.98 (1.44)^{a}	10.37 (3.27)^{a}	29.57 (8.05)^{a}	55 (43.3%)^{a}
p	0.021**	0.050**	0.912**	0.113**	0.015**	0.060***

* Student t-test ** Anova with Tukey post-hoc test. *** Pearson's chi-squared test. **** Fisher's exact test. For each variable. the same letters comparing lines do not differ significantly.
